# Short-term/long-term prognosis with or without beta-blockers in patients without heart failure and with preserved ejection fraction after acute myocardial infarction: a multicenter retrospective cohort study

**DOI:** 10.1186/s12872-022-02631-8

**Published:** 2022-04-26

**Authors:** Xue-song Wen, Rui Luo, Jie Liu, Qin Duan, Shu Qin, Jun Xiao, Dong-Ying Zhang

**Affiliations:** 1 Department of Cardiovascular Medicine, The First Affiliated Hospital of Chongqing Medical University, No. 1, Youyi Road, Chongqing, 400016 China; 2 Department of Cardiovascular Medicine, The First Branch of the First Affiliated Hospital of Chongqing Medical University, Chongqing, 400016 China; 3 Department of Cardiovascular Medicine, Chongqing University Center Hospital, No. 1, Jiankang Road, Yuzhong District, Chongqing, 400014 China

**Keywords:** Beta-blockers, Acute myocardial infarction, Heart failure, Left ventricular ejection fraction

## Abstract

**Background:**

The role of beta-blockers in acute myocardial infarction patients without heart failure and with preserved left ventricular ejection fraction (LVEF ≥ 50%) is unknown. Our study aimed to retrospectively analyze the associations of beta-blockers on such patients.

**Methods:**

This is a multicenter, retrospective study. After screening 5,332 acute myocardial infarction patients, a total of 2519 patients without heart failure and with LVEF ≥ 50% were included. The patients were divided into two groups: the prescribed (n = 2049) and unprescribed (n = 470) beta-blockers group. The propensity score inverse probability treatment weighting was used to control confounding factors. We analyzed the associations between beta-blockers and outcomes in the short-term (1-year) and long-term (median, 3.61 years).

**Results:**

The primary outcome was all-cause mortality. The secondary outcomes were all-cause rehospitalization, cardiac death, recurrent myocardial infarction, new-onset heart failure rehospitalization. This study shows no statistically significant association between discharged with beta-blockers and all-cause mortality, either in the short-term [IPTW Adjusted, HR 1.02; 95%CI 0.43–2.40; *P* = 0.966] or long-term [IPTW Adjusted, HR 1.17; 95%CI 0.70–1.94; *P* = 0.547]. Discharged with beta-blockers was significantly associated with a reduced risk of short-term recurrent myocardial infarction [IPTW Adjusted, HR 0.44; 95%CI 0.20–0.97; *P* = 0.043], but there was no long-term relationship [IPTW Adjusted, HR 1.11; 95%CI 0.61–2.03; *P* = 0.735]. Other outcomes, such as new-onset heart failure rehospitalization and all-cause rehospitalization, were not observed with meaningful differences in either the short- or long-term. The results of sensitivity analysis were consistent with this.

**Conclusions:**

Beta-blockers might be associated with a reduced risk of recurrent myocardial infarction in patients without heart failure and with preserved left ventricular ejection fraction after acute myocardial infarction, in the short term. Beta-blockers might not be related to all-cause mortality in those patients, either in the short-term or long-term.

*Clinical trial registration* Influence of Beta-blockers on Prognosis in Patients with Acute Myocardial Infarction Complicated with Normal Ejection Fraction, NCT04485988, Registered on 24/07/2020. Retrospectively registered.

**Supplementary Information:**

The online version contains supplementary material available at 10.1186/s12872-022-02631-8.

## Introduction

Some milestone studies such as BHAT (The Beta-blocker Heart Attack Trial), established the conventional use of beta-blockers after acute myocardial infarction (AMI) was published in the 1980s [1, 2]. The beta-blockers then become a central component of pharmacological treatment for AMI. A systematic review and meta-regression analysis showed that all the research evidence about beta-blockers after AMI was mainly from patients who did not receive reperfusion treatment and secondary prevention therapy [3].

With the widespread prevalence of reperfusion treatment and secondary prevention therapy, it is questionable whether beta-blockers would provide a survival benefit for all patients with AMI. The randomized controlled trial (RCT) which explored the prognostic role of beta-blockers in AMI patients noted that early intravenous metoprolol followed by four weeks of oral metoprolol did not contribute to the short-term survival prognosis (first discharge or day 28) in AMI patients, but no long-term results [4]. In addition, a meta-analysis (sixty trials, n = 102,003) suggested beta-blockers had no all-cause mortality benefit for patients with AMI during the period of reperfusion era (> 50% undergoing reperfusion and/or receiving aspirin/statin) [5]. All the evidence implied that beta-blockers might not lower the risk of all-cause mortality in AMI patients when treated with timely revascularization and secondary prevention therapy.

The role of beta-blockers in AMI patients without heart failure (HF) and with preserved left ventricular ejection fraction (LVEF) was unknown. In previous studies, the controversy regarding the associations of beta-blockers mainly exists in AMI patients with or without HF, LVEF normal (LVEF ≥ 40%) or reduce (LVEF < 40% or LVEF < 30%) after AMI [6–8]. The available evidence supporting the utilization of beta-blockers was in HF patients with LVEF < 40% [9]. There are no definite conclusions from RCTs on the prognostic value of beta-blockers in AMI patients without HF and with preserved LVEF, and the present guidelines or consensus were mainly based on other types of experiments, which caused the AMI patients to be prescribed beta-blockers ad infinitum regardless of HF and regardless of LVEF [10].

To our knowledge, this is the first study regarding the prognostic associations of beta-blockers on AMI patients without HF and with preserved ejection fraction (LVEF ≥ 50%). Our study aimed to retrospectively analyze the associations of beta-blockers on such patients and to infer whether beta-blockers changed the survival benefit of the patients.

## Methods

The baseline characteristics of the AMI patients were collected through the electronic medical record. The epidemiological data, risk factors, comorbidities, examination results, treatments, and prescribed medication information of the patients were recorded. During follow-up, the information on patient survival status and hospitalization events was collected through telephone interviews and medical documents.

This study analyzed the short-term and long-term associations of beta-blockers; analyzed the relationship between beta-blockers and all-cause death in different subgroups; and further validated the short-term and long-term associations of beta-blockers by propensity score matching in the sensitivity analysis. Finally, the study also explored the relationship between LVEF and all-cause death by a restrictive cubic spline curve.

The study was a multicenter, retrospective, observational registry project with clinicaltrials.gov identifier NCT04485988 (registered on 24/07/2020). We observed the Declaration of Helsinki guidelines. All study procedures were approved by the ethics committee of the first affiliated hospital of Chongqing medical university (approval number 2020-607).

### Definitions

The primary outcome was all-cause mortality. The secondary outcomes were all-cause rehospitalization, cardiac death, recurrent myocardial infarction, new-onset HF rehospitalization, and major adverse cardiovascular events (MACE, composite endpoint event of cardiac death, recurrent myocardial infarction, new-onset HF rehospitalization). AMI is defined by the elevation of serum markers of myocardial injury at least twice their upper limit of normal (creatine kinase isoenzyme or troponin I), ST-segment elevation or decrease in at least two contiguous leads greater than 0.1 mv, and pathological Q waves. Cardiac death was defined as death due to fatal AMI, HF, and death that cannot be attributed to non-cardiac causes. HF was defined as patients with a history of HF or discharged with symptoms of HF. LVEF is measured by the Simpson method of cardiac ultrasound, which is determined by the last measurement taken during hospitalization. Patients without HF and with preserved ejection fraction after AMI were defined as those with no previous history of HF and no symptoms of HF before discharge, as well as a LVEF ≥ 50%. Other percutaneous coronary intervention (PCI) includes delayed PCI and rescue PCI. Delayed PCI was defined as the time from symptom onset to PCI treatment longer than 72 h.

### Statistical analysis

Continuous variables were presented as mean ± standard deviation (SD) or median (interquartile range). Categorical variables were expressed as frequencies and percentages. Continuous variables were compared by using the independent samples T-test and the Mann–Whitney U-test. Categorical variables were tested by using the Chi-square test and Fisher's exact Chi-square test. Considering the number and complexity of variables in this study, as well as the fact that multivariate Cox adjustment is limited by the number of adjusted variables and outcome events, propensity score inverse probability treatment weighting (IPTW) was used to control for confounding factors. The propensity score can represent the associations of multiple confounding factors in a combined propensity score. IPTW is based on the propensity score and uses the principle of the standardization method to make the distribution of propensity scores consistent across groups so that the associations of confounding factors can be eliminated. Univariate Cox regression and propensity score IPTW correction were used to determine the association between beta-blockers and outcomes. Kaplan–Meier curves were used to assess prognostic differences between the two groups. The R Statistical Package, version 4.0.2 (R Development Team, Vienna, Austria), and IBM SPSS Statistics 26.0 software (SPSS, Chicago, IL, USA) were used for all statistical analyses. P (two-tailed) value less than 0.05 was considered statistically significant.

### Study population

**Fig. 1:**
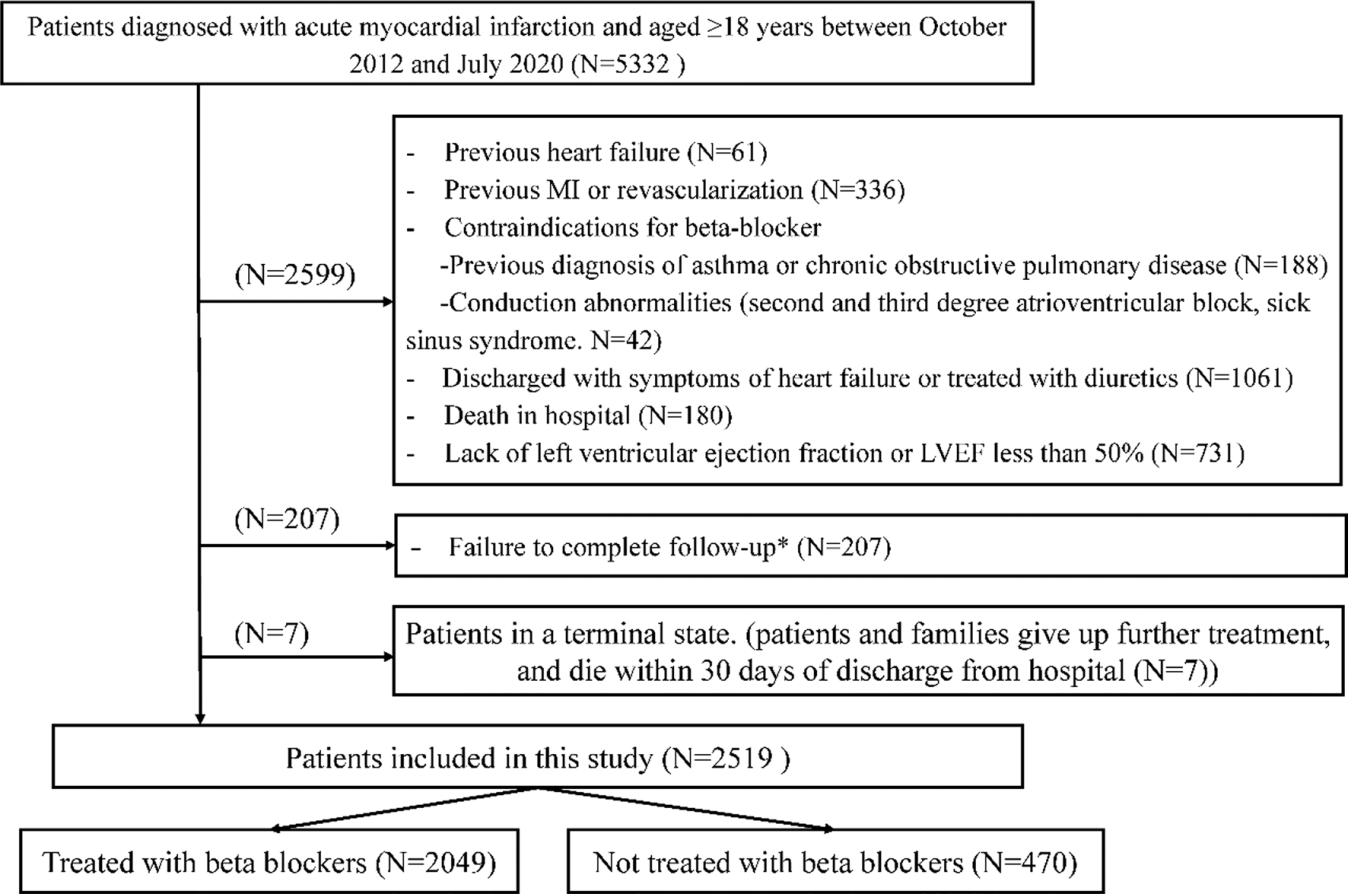
Flow diagram of patients recruitment. *207 patients with AMI were lost to follow-up, of which 168 patients could not be contacted again and 39 patients refused to communicate when contacted by phone

Patients with a definite diagnosis of AMI and aged ≥ 18 years were collected continuously from October 1, 2012, to July 31, 2020, and a total of 5,332 patients were recruited into the study. Patients with a history of HF, AMI, and reperfusion therapy, patients with contraindications to beta-blocker use (including chronic obstructive pulmonary disease (COPD), asthma, pacemaker implantation, second-degree/third-degree atrioventricular block, and sick sinus node syndrome), patients without information on LVEF value or with LVEF < 50%, lack of discharge prescription information or prescribed diuretics, and in-hospital death were excluded from the study. During the follow-up period, 207 patients were lost and 7 patients were in a terminal state (died within 30 days of discharge), both of which were also excluded. Ultimately, 2519 patients were included in the cohort study, 2049 of whom were prescribed beta-blockers at discharge and 470 were not (Fig. 1).

## Results

### Clinical characteristics

Of the 2519 patients included, 2049 were prescribed beta-blockers (2049/2519, 81.3%) and 470 were not (470/2519, 18.7%). Patients without beta-blockers were older, had more frequent inferior/posterior AMI, higher LVEF value, more comorbid history of stroke or atrial fibrillation, generally lower admission heart rate and blood pressure, lower body mass index, lower HbA1c, lower eGFR, lower low-density lipoprotein, lower blood urea nitrogen, lower creatine kinase isoenzyme MB, fewer of them were treated with PCI and reperfusion therapy, and fewer treated with Clopidogrel/Ticagrelor and angiotensin-converting enzyme inhibitor (ACEI)/ angiotensin receptor blocker (ARB)/ angiotensin receptor enkephalin inhibitor (ARNI) (Table 1).

**Table 1:** Baseline characteristics of patients discharged with beta-blockers and without beta-blockers

Variables	Discharged with beta-blockers (N = 2049)	Discharged without beta-blockers (N = 470)	*P* value
*Baseline characteristics*			
Age, years	62.0 (52.0–70.0)	64.0 (55.0–73.0)	< 0.001
Age ≥ 75 years	295 (14.4%)	101 (21.5%)	< 0.001
Male sex	1624 (79.3%)	378 (80.4%)	0.613
Body mass index (kg/m^2^)	24.3 (22.4–26.6)	23.4 (21.5–25.7)	< 0.001
Body mass index ≥ 30.0	96 (4.9%)79	17 (3.7%)	0.326
Onset time	11.0 (4.0–48.0)	12.0 (4.0–48.0)	0.869
*Risk factors–-no, %*			
Hypertension	1147 (56.0%)	249 (53.0%)	0.258
Diabetes mellitus	643 (31.4%)	138 (29.4%)	0.407
Hyperlipidemia	424 (20.7%)	80 (17.0%)	0.074
Cigarette smoking	1337 (65.3%)	316 (67.2%)	0.420
Family history of CAD	120 (5.9%)	23 (4.9%)	0.507
*Medical history–-no, %*			
Previous CAD	61 (3.0%)	10 (2.1%)	0.357
Chronic kidney disease	83 (4.1%)	17 (3.6%)	0.793
Previous stroke or TIA	123 (6.0%)	45 (9.6%)	0.007
Atrial fibrillation	70 (3.4%)	29 (6.2%)	0.005
Peripheral vascular disease	21 (1.0%)	2 (0.4%)	0.288
Malignant tumor	39 (1.9%)	8 (1.7%)	1.000
*Myocardial infarction characteristics–-no, %*			
STEMI	1324 (64.6%)	297 (63.2%)	0.558
Anterior MI	715 (54.0%)	76 (25.6%)	< 0.001
Inferior/ Posterior MI	639 (48.3%)	221 (74.4%)	< 0.001
Other sites MI	214 (16.2%)	41 (13.8%)	< 0.001
Killip class ≥ II	226 (11.0%)	56 (11.9%)	0.571
Coronary angiography	1939 (94.6%)	434 (92.3%)	0.062
Thrombolytic therapy	48 (2.3%)	13 (2.8%)	0.617
PTCA therapy	63 (3.1%)	18 (3.8%)	0.386
PCI therapy	1673 (81.6%)	331 (70.4%)	< 0.001
PCI within 72 h	1128 (55.1%)	236 (50.2%)	0.065
Other PCI	545 (26.6%)	95 (20.2%)	0.004
Coronary artery bypass grafting	2 (0.1%)	2 (0.4%)	0.160
Timely reperfusion therapy	721 (35.2%)	152 (32.3%)	0.259
Total revascularization	1685 (82.2%)	333 (70.9%)	< 0.001
*Presenting characteristics*			
Admission heart rate (beats/min)	79.0 (70.0–90.0)	70.0 (60.0–78.0)	< 0.001
Heart rate > 110 beats/min	69 (3.4%)	6 (1.3%)	0.015
Admission SBP (mm Hg)	131.0 (115.0–149.0)	123.5 (107.0–141.0)	< 0.001
Admission SBP < 90 mm Hg	58 (2.8%)	29 (6.2%)	0.001
Admission DBP (mm Hg)	79.0 (69.0–90.0)	73.0 (64.0–82.0)	< 0.001
Peak CK-MB (ug/L)	16.5 (3.6–55.6)	13.6 (3.0–48.8)	0.039
Peak troponin-I (ng/mL)	1.24 (0.23–7.07)	1.32 (0.22–6.81)	0.986
HbA1c (%)	6.1 (5.7–7.0)	5.9 (5.6–6.6)	0.004
Blood urea nitrogen (mmol/L)	5.4 (4.5–6.7)	5.8 (4.6–7.1)	0.001
Creatinine (umol/L)	74.0 (63.0–88.0)	76.0 (65.0–88.5)	0.101
eGFR (mL/min/1.73m^2^)	94.6 (76.6–107.6)	92.0 (73.5–103.8)	0.005
eGFR < 60 mL/min/1.73m^2^	246 (12.2%)	52 (11.2%)	0.634
Low-density lipoprotein (mg/dl)	2.75 (2.19–3.36)	2.59 (2.06–3.08)	< 0.001
LVEF (%)	58.0 (55.0–62.0)	60.0 (56.0–63.0)	0.001
Cardiac aneurysm	28 (1.8%)	6 (1.6%)	1.000
*Concomitant medication–-No, %*			
Aspirin	1905 (93.0%)	425 (90.4%)	0.065
Clopidogrel/ticagrelor	2024 (98.8%)	455 (96.8%)	0.006
DAPT	1888 (92.2%)	413 (87.9%)	0.004
Statin	2023 (98.7%)	463 (98.5%)	0.656
ACEI/ ARB/ ARNI	1528 (74.6%)	264 (56.2%)	< 0.001
Oral anticoagulant	34 (1.7%)	11 (2.3%)	0.333

CAD, coronary atherosclerotic heart disease; TIA, transient ischemic attacks; STEMI, ST-segment elevation myocardial infarction; MI, myocardial infarction; PTCA, percutaneous transluminal coronary angioplasty; PCI, percutaneous coronary intervention; SBP, systolic blood pressure; DBP, diastolic blood pressure; CK-MB, creatine kinase isoenzyme MB; eGFR, estimated glomerular filtration rate; LVEF, left ventricular ejection fraction; DAPT, dual antiplatelet therapy; ACEI, angiotensin-converting enzyme inhibitor; ARB, angiotensin receptor blocker; ARNI, angiotensin receptor enkephalin inhibitor

The types of prescribed beta-blockers in this study included metoprolol extended-release tablets (1705, 83.2%), metoprolol pills (34, 1.7%), bisoprolol tablets (292, 14.3%), and others (including carvedilol and atenolol, 18, 0.9%). The dose of prescribed beta-blockers is expressed by percentage (%) of dose = discharged prescribed beta-blocker dose/target dose. 101 patients (4.9%) were prescribed doses of 0–12.5% target dose, 727 patients (35.5%) were prescribed doses of 12.5–25% target dose, 1033 patients (50.4%) were prescribed doses of 25–50% target dose, and 188 patients (9.2%) were prescribed doses greater than 50% target dose.

In our study, 207 patients with AMI were lost to follow-up, of which 168 patients could not be contacted again and 39 patients refused to communicate when contacted by phone.

### Outcomes

**Fig. 2:**
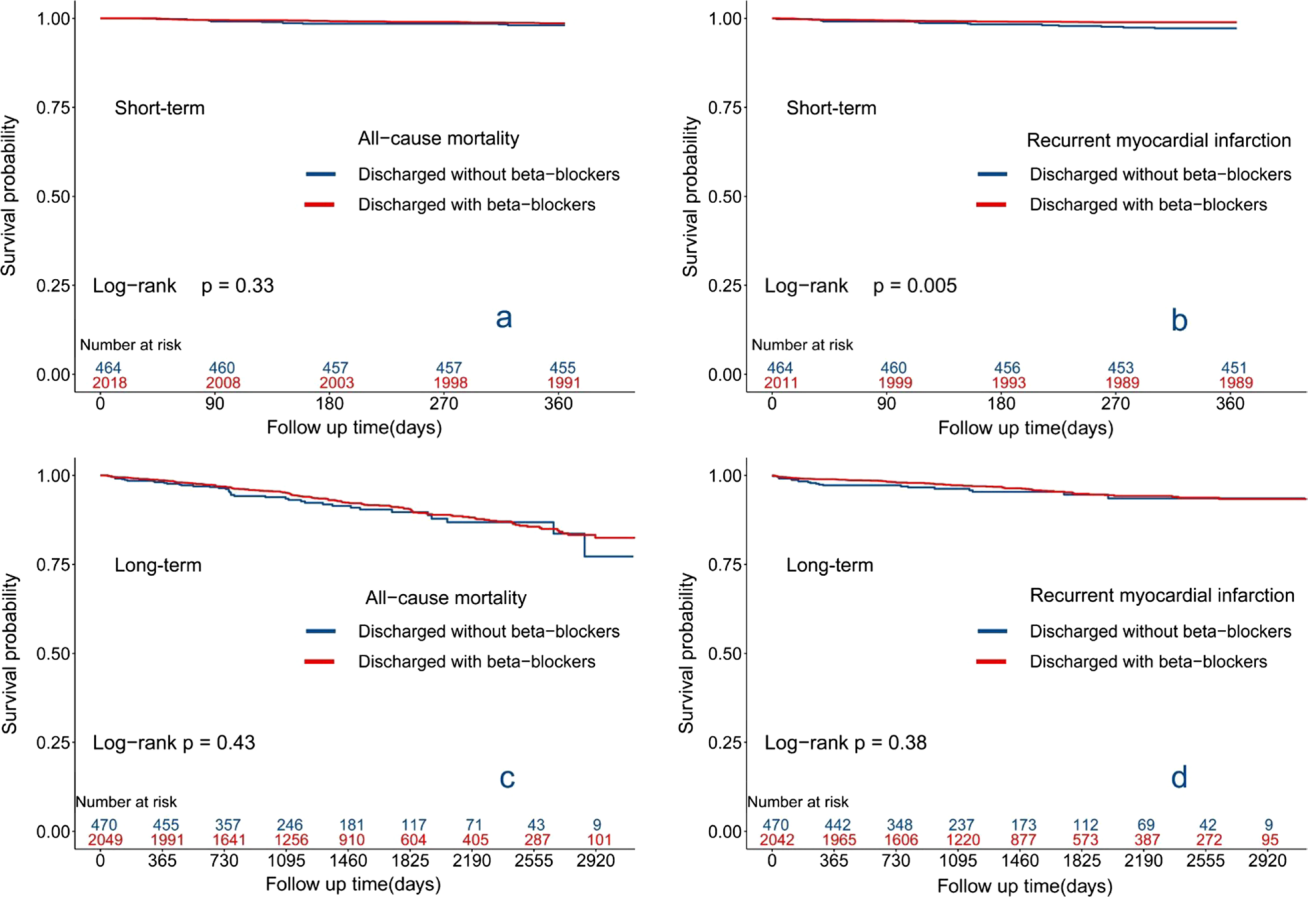
Kaplan–Meier survival estimates. **a**, **b** Demonstrate the short-term (follow-up, one year, N = 2482) association between beta-blockers and all-cause mortality/recurrent myocardial infarction. A log-rank test was used. The results suggest that there was no statistically significant association between discharge prescription of beta-blockers and risk of all-cause mortality and a statistically significant association between discharge prescription of beta-blockers and reduced risk of recurrent myocardial infarction. **c**, **d** Demonstrate the long-term (median follow-up, 3.75 years, N = 2519) association between beta-blockers and all-cause mortality/recurrent myocardial infarction. A log-rank test was used. The results suggest that there was no statistically significant association between discharge prescription of beta-blockers and risk of all-cause mortality/ recurrent myocardial infarction. IPTW correction: based on propensity score with inverse probability treatment weighting (IPTW), adjusted for factors as described in Table 2

We followed the enrolled 2519 patients for a median of 3.61 (2.12–5.27) years. We analyzed the short-term/long-term associations of prescribed beta-blockers. In the short-term, there was an association between discharge-prescribed beta-blockers and reduced risk of recurrent myocardial infarction [univariate COX regression, HR 0.39, 95%CI 0.20–0.77, *P* = 0.007; IPTW, HR 0.44, 95%CI 0.20–0.97, *P* = 0.043]. For other outcomes, such as all-cause mortality, cardiac death, all-cause rehospitalization, new-onset HF rehospitalization, and MACE, there was no significant association between discharged with beta-blockers and each of them (Table 2; Fig. 2).

**Table 2:** Association of beta-blockers with outcomes in the short-term

Variables	With beta-blockers	Without beta-blockers	Crude HR	*P* value	Adjusted HR^a^	*P* value
	N = 2049	N = 470				
	No. of patients with event (n, %)	No. of patients with event (n, %)	(95% CI)		(95% CI)	
One year after discharge (n = 2482)						
All-cause mortality	27/2018 (1.3%)	9/464 (1.9%)	0.69 (0.32–1.46)	0.330	1.02 (0.43–2.40)	0.966
Rehospitalization for any reason	299/1977 (15.1%)	88/459 (19.2%)	0.76 (0.60–0.97)	0.026	0.83 (0.60–1.15)	0.258
Cardiac death	15/2018 (0.7%)	5/464 (1.1%)	0.69 (0.25–1.89)	0.469	1.25 (0.37–4.25)	0.719
Rehospitalization for MI	22/2011 (1.1%)	13/464 (2.8%)	0.39 (0.20–0.77)	0.007	0.44 (0.20–0.97)	0.043
Rehospitalization for HF	34/2015 (1.7%)	9/464 (1.9%)	0.87 (0.42–1.82)	0.712	2.14 (0.98–4.69)	0.057
MACE	61/2009 (3.0%)	25/464 (5.4%)	0.56 (0.35–0.89)	0.014	0.80 (0.45–1.41)	0.434

This study analyzed the relationship between beta-blockers and short-term outcomes, using univariate COX regression and propensity score IPTW

MI, myocardial infarction; HF, heart failure; MACE, major adverse cardiovascular events; HR, hazard ratio; NA, not applicable; ref, reference

^a^Correction was performed using propensity score inverse probability treatment weighting (IPTW), included variables were sex, age, time of onset, LVEF, type of myocardial infarction, admission heart rate, admission systolic blood pressure, admission diastolic blood pressure, body mass index (BMI), site of myocardial infarction, Killip ≥ II, history of hypertension, history of diabetes mellitus, history of chronic kidney disease, history of coronary artery disease (CAD), family history of CAD, history of stroke, history of peripheral vascular disease, history of hyperlipidemia, history of smoking, history of tumor, atrial fibrillation, coronary angiography, PTCA therapy, PCI therapy, thrombolytic therapy, type of PCI, timely reperfusion therapy, total reperfusion therapy, CKMB, TnI, HbA1c, blood urea nitrogen, creatinine, eGFR, LDL-c, cardiac aneurysm, anticoagulants, aspirin, clopidogrel/ticagrelor, dual antiplatelet therapy, statins, ACEI/ARB/ARNI

In the long term, all-cause mortality occurred in 39 (39/470, 8.3%) and 166 (166/2049, 8.1%) patients in the unprescribed and prescribed groups, separately. Univariate Cox regression analysis showed no difference in the risk of all-cause death [HR 0.87; 95%CI 0.61–1.23; *P* = 0.430], nor did it differ after correction for IPTW [HR 1.17; 95%CI 0.70–1.94; *P* = 0.547]. There were 20 cases of recurrent myocardial infarction in the unprescribed group (20/470, 4.3%), and 76 cases in the prescribed group (76/2042, 3.7%). The results suggested no obvious relationship between discharged with beta-blockers and the risk of recurrent myocardial infarction [univariate COX regression, HR 0.80, 95%CI 0.49–1.31, *P* = 0.381; IPTW, HR 1.11, 95%CI 0.62–2.03, *P* = 0.735]. Other endpoint events, including all-cause rehospitalization, new-onset HF rehospitalization, cardiac death, MACE, were not statistically significantly associated with beta-blockers (Table 3; Fig. 2).

**Table 3:** Association of beta-blockers with outcomes in the long-term

Events	Discharged with beta-blockers (N = 2049)	Discharged without beta-blockers (N = 470)	*P* value
*All-cause mortality*			
No. of patients with event^a^	166/2049 (8.1%)	39/470 (8.3%)	
Unadjusted HR (95% CI)	0.87 (0.61–1.23)	1.00 (ref)	0.430
Adjusted HR (95% CI)^a^	1.17 (0.70–1.94)	1.00 (ref)	0.547
*Rehospitalization for any reason*			
No. of patients with event	718/2008 (35.8%)	192/465 (41.3%)	
Unadjusted HR (95% CI)	0.76 (0.65–0.90)	1.00 (ref)	< 0.001
Adjusted HR (95% CI)	0.94 (0.73–1.20)	1.00 (ref)	0.605
*Cardiac death*			
No. of patients with event	100/2049 (4.9%)	24/470 (5.1%)	
Unadjusted HR (95% CI)	0.85 (0.55–1.33)	1.00 (ref)	0.485
Adjusted HR (95%CI)	1.36 (0.80–2.33)	1.00 (ref)	0.254
*Recurrent myocardial infarction*			
No. of patients with event	76/2042 (3.7%)	20/470 (4.3%)	
Unadjusted HR (95% CI)	0.80 (0.49–1.31)	1.00 (ref)	0.381
Adjusted HR (95%CI)	1.11 (0.61–2.03)	1.00 (ref)	0.735
*Rehospitalization for heart failure*			
No. of patients with event	124/2046 (6.1%)	25/470 (5.3%)	
Unadjusted HR (95% CI)	1.06 (0.69–1.62)	1.00 (ref)	0.808
Adjusted HR (95% CI)	1.63 (0.98–2.70)	1.00 (ref)	0.073
*MACE*			
No. of patients with event	230/2040 (11.3%)	53/470 (11.3%)	
Unadjusted HR (95% CI)	0.91 (0.68–1.23)	1.00 (ref)	0.549
Adjusted HR (95%CI)	1.35 (0.93–1.98)	1.00 (ref)	0.116

This study analyzed the relationship between beta-blockers and long-term outcomes. Univariate Cox analysis and propensity score IPTW corrected was performed (N = 2519)

AMI, acute myocardial infarction; LVEF, left ventricular ejection infraction; MACE, major adverse cardiovascular events; HR, hazard ratio; ref, reference

^a^Correction was performed using propensity score inverse probability treatment weighting (IPTW), included variables were sex, age, time of onset, LVEF, type of myocardial infarction, admission heart rate, admission systolic blood pressure, admission diastolic blood pressure, body mass index (BMI), site of myocardial infarction, Killip ≥ II, history of hypertension, history of diabetes mellitus, history of chronic kidney disease, history of coronary artery disease (CAD), family history of CAD, history of stroke, history of peripheral vascular disease, history of hyperlipidemia, history of smoking, history of tumor, atrial fibrillation, coronary angiography, PTCA therapy, PCI therapy, thrombolytic therapy, type of PCI, timely reperfusion therapy, total reperfusion therapy, CKMB, TnI, HbA1c, blood urea nitrogen, creatinine, eGFR, LDL-c, cardiac aneurysm, anticoagulants, aspirin, clopidogrel/ticagrelor, dual antiplatelet therapy, statins, ACEI/ARB/ARNI

Discharged with beta-blockers might only reduce the risk of short-term recurrent myocardial infarction in patients without HF and with preserved LVEF after AMI.

### Subgroups analysis

**Fig. 3:**
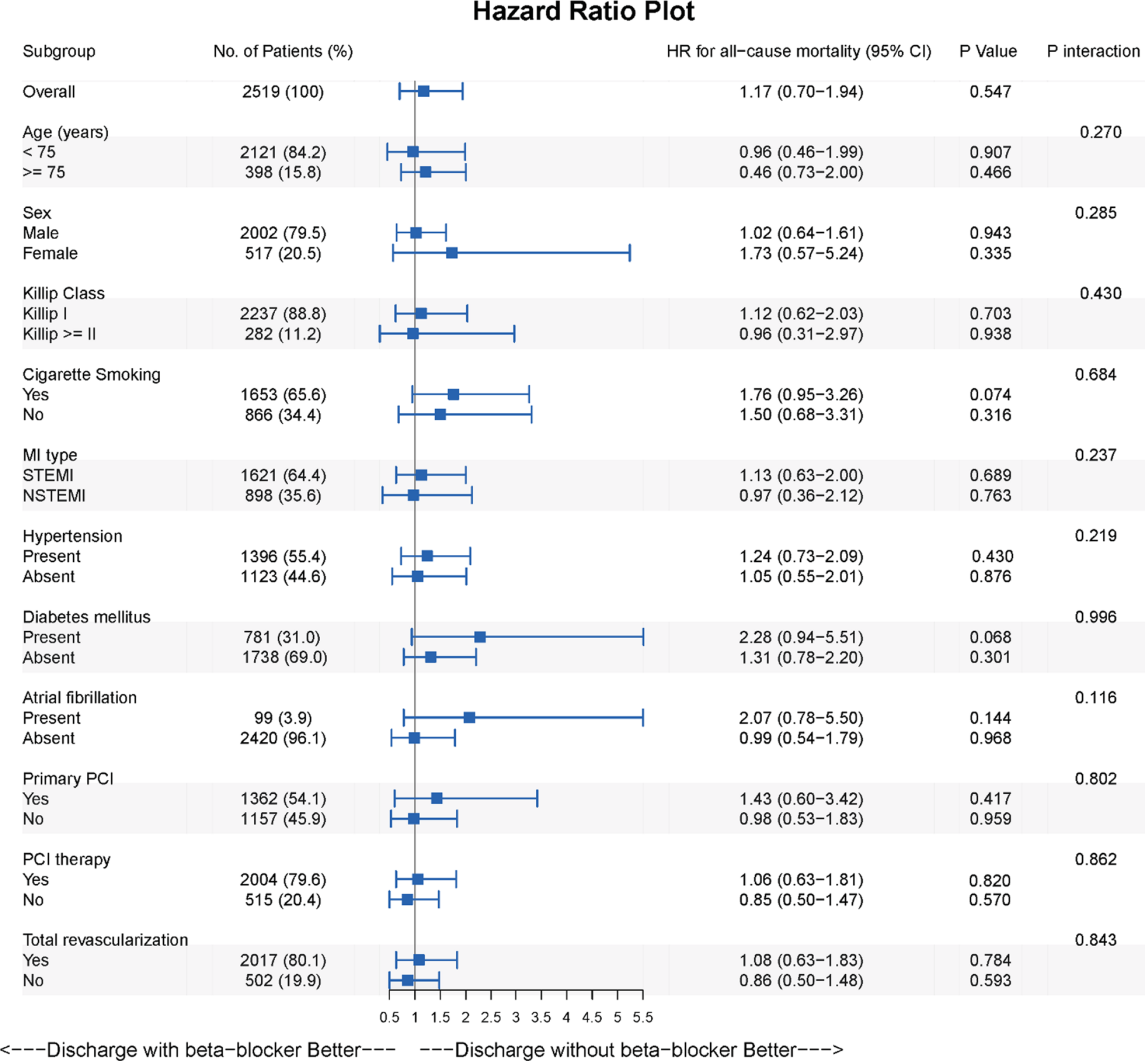
Subgroups analysis. The association of beta-blockers with all-cause mortality was analyzed in different subgroups (N = 2519). In this study, no statistically significant association between beta-blockers and subgroups was observed, and there was no interaction. The P-values were adjusted with propensity score inverse probability treatment weighting (IPTW), and the adjusted factors are shown in Table 2

To gain further insight and understanding of the role of beta-blockers in AMI patients without HF and with preserved LVEF, subgroup analyses were conducted by age (age < 75 vs. ≥ 75), sex, Killip class (I class vs. > I class), type of MI (STEMI vs. NSTEMI), smoke, hypertension, diabetes, atrial fibrillation, primary PCI therapy, PCI treatment, and reperfusion therapy. Based on propensity score with IPTW, the results indicated that among the subgroups, there was no significant difference in the risk of all-cause mortality between the prescribed and unprescribed groups; discharged with beta-blockers might not be related to a reduced risk of all-cause mortality. The study noted no interaction between subgroups and prescribed/unprescribed beta-blockers for all-cause mortality (Fig. 3).

## Sensitivity analysis

### Propensity score matching

We performed logit regression with prescribed beta-blockers as the dependent variable and each variable in Table 1 as a covariate, and took nearest neighbor matching without replacement for 1:1 matching, with a caliper value of 0.02. The absolute standardized mean difference (SMD) values for all variables were less than 0.1, indicating a well-paired match. The study was ultimately successful in matching 848 patients (discharged with beta-blockers, N = 424; discharged without beta-blockers, N = 424, Additional file 1: Table S1).

### Outcomes

We analyzed the associations between the beta-blockers and endpoint events in the short-term (1-year) and long-term (median, 3.75 years). The results showed, at one-year post-discharge (n = 835), recurrent myocardial infarction was observed in 12 cases (12/420, 2.9%) in the unprescribed group and 1 case (1/415, 0.2%) in another group, beta-blockers might be associated with a lower risk of recurrent myocardial infarction [IPTW correction, HR 0.08; 95% CI 0.01–0.59; *P* = 0.014] but not the risk of all-cause death [IPTW correction, HR 1.08; 95% CI 0.40–2.94; *P* = 0.875] (Additional file 1: Table S2). In the long-term, all-cause death was observed in 33 cases (33/424, 7.8%) in the unprescribed group and 35 cases (35/424, 8.3%) in another group, with no significant difference in the risk of all-cause death as indicated by univariate Cox regression analysis, and after IPTW adjustment [HR 0.96; 95%CI 0.60–1.55; *P* = 0.872], no significant difference remained. Other endpoints, such as all-cause rehospitalization, recurrent myocardial infarction, new-onset HF rehospitalization, cardiac death, and MACE, showed no remarkable distinction between the two groups (Additional file 1: Table S3).

### Supplementary analysis

We used a restricted cubic splines curve (RCS curve) to flexibly model and visualize the association of LVEF values with all-cause mortality in AMI patients without HF and with LVEF ≥ 50%. The results (n = 2519, three knots) suggested that LVEF values were statistically significant overall (*P* = 0.005) and had a non-linear association with all-cause mortality (P-Nonlinear = 0.043), adjusted using propensity score IPTW. Not all LVEF values were statistically significantly associated with all-cause mortality in this study. An association between LVEF values and reduced risk of all-cause mortality existed only when 50% ≤ LVEF ≤ 59%. Overall, when LVEF ≥ 50%, hazard ratio values were almost always below 1.0, which implies a lower risk of all-cause mortality, and LVEF ≥ 50% might be a protective factor for all-cause mortality in these patients (Additional file 1: Fig. S1).

## Discussion

The present study was designed to detect the appropriate use of beta-blockers in AMI patients without HF and with LVEF ≥ 50%. We found beta-blockers might reduce the risk of recurrent myocardial infarction in short-term (1-year); beta-blockers could not improve the short and long-term risk of all-cause mortality, all-cause rehospitalization, hospitalization for HF, and cardiac death in those patients; all-cause mortality might not be associated with beta-blockers in all subgroups; LVEF values ≥ 50% might be a protective factor for all-cause mortality.

Recommendations for the beta-blockers in AMI patients differ from the guidelines. The European Society of Cardiology (ESC 2015/2017) recommend that long-term application of beta-blockers is required in patients with STEMI (IIa, B) or NSTEMI (I, A) only if combined with a reduced left ventricular systolic function [11, 12], whereas the American College of Cardiology/American Heart Association (ACC 2013/AHA 2014) recommend that patients with STEMI (I, B) or NSTEMI (IIa, C) should be administered consistently after discharge, regardless of ejection fraction [13, 14]. Such disparate recommendations pose a problem for the individualized use of beta-blockers in AMI patients, which caused AMI patients to be prescribed beta-blockers endlessly irrespective of HF and irrespective of LVEF.

To our knowledge, this is the first study regarding the prognostic associations of beta-blockers on AMI patients without HF and with preserved ejection fraction (LVEF ≥ 50%). We found that the beta-blockers failed to lower the risk of all-cause mortality, in the short and long term. Review of previous relevant studies. AMI patients without HF (n = 30177, no LVEF information available) did not benefit from beta-blockers prescribed at discharge, in terms of cardiovascular prognosis, according to Holt et al. [15]. In a multicenter prospective cohort study (223 centers, n = 2679), prescribed beta-blockers at discharge might not associate with lower one-year mortality in AMI patients without HF and LVEF > 40% (enrolled patients with COPD/previous AMI/previous PCI/previous CABG) [6]. Also, the study noted prescribed beta-blockers at discharge did not reduce the risk of all-cause mortality within one year in AMI patients without HF or LVEF > 30% (n = 91895) [7]. Besides, the result of a meta-analysis suggested the application of beta-blockers did not contribute to a significant reduction in the risk of all-cause mortality in AMI patients without HF or with LVEF > 40% (in reality, some patients with HF or LVEF < 40%) [16]. Previous related studies have found similar results in different populations. In contrast to the above studies, the present study population was distinct, our study strictly determined the LVEF threshold at 50% and all AMI patients without HF. Also this study with stringent inclusion and exclusion standards, the possibility of confounding can be reduced.

There are some dissenting opinions about the role of beta-blockers. Irrespective of LVEF, prescribed beta-blockers at discharge reduced the one-year risk of all-cause mortality by 29% in AMI patients (n = 2592; LVEF < 40% accounted for 12.4%, n = 321) [17]. AMI Patients with reduced LVEF (LVEF ≤ 40%, n = 1670) and intermediate-range LVEF (40% < LVEF < 50%, n = 2904) had a reduced risk of MACE at one year, but the benefit was not present in AMI patients with preserved LVEF (LVEF ≥ 50%, n = 7626) [18]. Those two reports enrolled AMI patients with LVEF < 50% and AMI patients with HF, the benefit of beta-blockers may be derived from heart failure with reduced ejection fraction (HFrEF) patients [17, 18]. Our study did not cover patients with HF and with a longer follow-up period. A recent cohort study in Korea reported that treatment with beta-blockers for more than one year after discharge among AMI patients without HF resulted in a reduced risk of all-cause mortality (the LVEF information was not revealed) [8]. However, the secondary prevention medications in this study were used more frequently in patients treated with beta-blockers than those not, such as aspirin (94.9% vs. 69.7%, *P* < 0.001), clopidogrel (69.3% vs. 50.7%, *P* < 0.001), and statins (95.2% vs. 68.6%, *P* < 0.001). The benefits of beta-blockers observed in the study may be derived from other secondary prevention agents for coronary heart disease.

In our study, patients prescribed beta-blockers were more often treated with ACEI/ARB/ARNI, considering possible reasons: patients prescribed beta-blockers were younger, had fewer inferior or posterior wall myocardial infarctions, and had higher and more stable blood pressure on admission.

There are some likely reasons for the lack of prognostic benefit of beta-blockers observed among AMI patients without HF and with preserved LVEF. Firstly, studies have found beta-blockers acquire beneficial effects by reducing fatal arrhythmias, myocardial ischemia, and reinfarction, but patients with preserved LVEF may have less myocardial scarring and more surviving cardiomyocytes than AMI patients with reduced LVEF [18]. Secondly, sympathetic activity is enhanced after AMI. Beta-blockers may suppress cardiac remodeling by reducing oxygen consumption and prevent arrhythmias by slowing the heart rate. The rapid reperfusion therapy after AMI may suppress sympathetic activity to achieve a similar effect as beta-blockers [7]. In particular, the large-scale construction of chest pain centers in China has further shortened revascularization time. Thirdly, beta-blockers were essential in the treatment of HFrEF, but limited evidence supports their use in heart failure with mid-range ejection fraction (HFmrEF) or heart failure with preserved ejection fraction (HFpEF) [9]. To accurately determine the value of beta-blockers, we only choose AMI patients without HF and with preserved LVEF.

Investigating the role of beta-blockers in AMI patients without HF and with preserved ejection fraction is of great importance for the accurate treatment of patients with AMI. There are several randomized controlled trials that are exploring the role of beta-blockers in patients without reduced LVEF after AMI (NCT03596385, NCT03646357, NCT03498066, NCT03278509), and we expect that they will provide a high-quality reference for the use of beta-blockers in patients with AMI.

### Limitations

In this retrospective, observational study, although 5,332 patients were initially recruited, only 2519 patients were ultimately included in the cohort. The scientific validity of the study is limited by the sample size and the inherent failure to correct for unknown additional confounders. Second, all-cause mortality was a competing risk for recurrent myocardial infarction in our study [19, 20], and to some extent affects the evaluation of the utility of beta-blockers for recurrent myocardial infarction; future studies need to take this issue of competing risks into account. Third, our study is a retrospective study and it is difficult to obtain information about the use of beta-blockers over the entire follow-up period in patients with AMI. For example, whether the use of beta-blockers was regular? What was the dose used? Also, our findings are not generalizable and apply to a select subgroup of patients with AMI (without HF and with preserved LVEF).

## Conclusions

Prescribed beta-blockers might not be associated with all-cause mortality in AMI patients without HF and with preserved LVEF (LVEF ≥ 50%), either in the short or long term. However, beta-blockers might be associated with a reduced risk of recurrent myocardial infarction in those patients in the short term. Re-evaluation of the role of beta-blockers in those patients is still warranted.

## Supplementary Information

**Additional file 1: **Sensitivity analysis results and supplementary analysis results.

## Data Availability

The datasets used and analyzed during the current study are available from the corresponding author on reasonable request.

## Abbreviations

AMI: Acute myocardial infarction
HF: Heart failure
LVEF: Left ventricular ejection fraction
PSM: Propensity score matching
IPTW: Inverse probability of treatment weighting
MACE: Major cardiovascular adverse events
STEMI: St-segment elevation myocardial infarction
NSTEMI: Non-st-segment elevation myocardial infarction
PCI: Percutaneous coronary intervention
CK-MB: Creatine kinase isoenzyme MB
eGFR: Estimated glomerular filtration rate
ACEI: Angiotensin-converting enzyme inhibitor
ARB: Angiotensin receptor blocker
ARNI: Angiotensin receptor enkephalin inhibitor
HFmrEF: Heart failure with mid-range ejection fraction
HFpEF: Heart failure with preserved ejection fraction
