# Supplementary material for: Short-term/long-term prognosis with or without beta-blockers in patients without heart failure and with preserved ejection fraction after acute myocardial infarction: a multicenter retrospective cohort study
Source: BMC Cardiovasc Disord. 2022 Apr 26;22:193. doi: 10.1186/s12872-022-02631-8 (PMC9044853; doi:10.1186/s12872-022-02631-8)
Supplement: Supplementary file 1 — Additional file 1: Sensitivity analysis results and supplementary analysis results. [file 12872_2022_2631_MOESM1_ESM.docx]

**Table S1.** Baseline characteristics of patients after propensity score matching.

| **Variables** | Overall (N=848) | Discharged with beta-blockers (N=424) | Discharged without beta-blockers (N=424) | *P*-value |
| --- | --- | --- | --- | --- |
| **Baseline Characteristics** | | | | |
| Age (years) | 63.0 (54.0-72.0) | 63.0 (54.0-72.0) | 64.0 (54.0-72.0) | 0.586 |
| --Age ≥75 years | 151 (17.8%) | 66 (15.6%) | 85 (20.0%) | 0.106 |
| Male sex | 676 (79.7%) | 336 (79.2%) | 340 (80.2%) | 0.798 |
| Body mass index (kg/m^2^) | 23.9 (22.0-25.7) | 23.9 (22.0-25.9) | 23.7 (22.0-25.7) | 0.483 |
| --Body mass index ≥ 30.0 | 33 (3.9%) | 17 (4.0%) | 16 (3.8%) | 1.000 |
| Time of onset (hours) | 12.0 (5.0-72.0) | 13.0 (5.0-72.0) | 12.0 (4.0-48.0) | 0.368 |
| **Risk factors---No, %** | | | | |
| Hypertension | 443 (52.2%) | 220 (51.9%) | 223 (52.6%) | 0.891 |
| Diabetes mellitus | 251 (29.6%) | 125 (29.5%) | 126 (29.7%) | 1.000 |
| Hyperlipidemia | 159 (18.8%) | 84 (19.8%) | 75 (17.7%) | 0.482 |
| Cigarette Smoking | 568 (67.0%) | 280 (66.0%) | 288 (67.9%) | 0.609 |
| Family history of CAD | 48 (5.7%) | 26 (6.1%) | 22 (5.2%) | 0.656 |
| **Medical history---No, %** | | | | |
| Previous CAD | 19 (2.2%) | 10 (2.4%) | 9 (2.1%) | 1.000 |
| Chronic kidney disease | 31 (3.7%) | 16 (3.8%) | 15 (3.5%) | 1.000 |
| Previous stroke or TIA | 80 (9.4%) | 45 (10.6%) | 35 (8.3%) | 0.290 |
| Atrial fibrillation | 46 (5.4%) | 22 (5.2%) | 24 (5.7%) | 0.880 |
| Peripheral vascular disease | 3 (0.4%) | 1 (0.2%) | 2 (0.5%) | 1.000 |
| Malignant tumor | 13 (1.5%) | 7 (1.7%) | 6 (1.4%) | 1.000 |
| **Myocardial infarction characteristics---No, %** | | | | |
| STEMI | 520 (61.3%) | 257 (60.6%) | 263 (62.0%) | 0.724 |
| Anterior MI | 142 (16.7%) | 67 (15.8%) | 75 (17.7%) | 0.520 |
| Inferior/ Posterior MI | 371 (43.8%) | 183 (43.2%) | 188 (44.3%) | 0.782 |
| Other sites MI | 86 (10.1%) | 48 (11.3%) | 38 (9.0%) | 0.306 |
| Killip class ≥ II | 89 (10.5%) | 40 (9.4%) | 49 (11.6%) | 0.370 |
| Coronary angiography | 784 (92.5%) | 390 (92.0%) | 394 (92.9%) | 0.697 |
| Thrombolytic therapy | 21 (2.5%) | 11 (2.6%) | 10 (2.4%) | 1.000 |
| PTCA therapy | 29 (3.4%) | 14 (3.3%) | 15 (3.5%) | 1.000 |
| PCI therapy | 612 (72.2%) | 300 (70.8%) | 312 (73.6%) | 0.399 |
| --PCI within 72h | 438 (51.7%) | 215 (50.7%) | 223 (52.6%) | 0.631 |
| --Other PCI | 174 (20.5%) | 85 (20.0%) | 89 (21.0%) | 0.799 |
| CABG | 4 (0.5%) | 2 (0.5%) | 2 (0.5%) | 1.000 |
| Timely reperfusion therapy | 273 (32.2%) | 132 (31.1%) | 141 (33.3%) | 0.557 |
| Total revascularization | 616 (72.6%) | 302 (71.2%) | 314 (74.1%) | 0.397 |
| **Presenting characteristics** | | | | |
| Admission HR (beats/min) | 70.0 (62.0-80.0) | 71.0 (64.0-80.0) | 70.0 (61.0-80.0) | 0.109 |
| Heart rate > 110 beats/min | 11 (1.3%) | 3 (0.7%) | 8 (1.9%) | 0.224 |
| Admission SBP (mm Hg) | 125.0 (108.0-143.0) | 126.0 (108.0-144.0) | 123.5 (108.0-141.5) | 0.333 |
| Admission SBP < 90 mm Hg | 41 (4.8%) | 16 (3.8%) | 25 (5.9%) | 0.200 |
| Admission DBP (mm Hg) | 74.0 (64.3-84.0) | 75.0 (64.5-84.0) | 73.0 (64.5-82.0) | 0.183 |
| Peak CK-MB (ug/L) | 15.8 (3.5-44.4) | 15.6 (3.5-41.8) | 15.8 (3.5-47.3) | 0.727 |
| Peak troponin-I (ng/mL) | 1.25 (0.25-6.28) | 1.25 (0.23-5.81) | 1.30 (0.26-6.92) | 0.325 |
| HbA1c (%) | 6.1 (5.8-6.3) | 6.1 (5.8-6.4) | 6.1 (5.8-6.3) | 0.514 |
| Blood urea nitrogen (mmol/L) | 5.7 (4.6-6.9) | 5.6 (4.7-6.8) | 5.7 (4.6-6.9) | 0.809 |
| Creatinine (umol/L) | 75.0 (65.0-87.0) | 75.0 (64.0-87.0) | 75.0 (66.0-87.0) | 0.567 |
| eGFR (mL/min/1.73m^2^) | 92.9 (75.8-103.6) | 92.8 (76.8-103.2) | 92.9 (74.4-103.9) | 0.722 |
| eGFR < 60 mL/min/1.73m^2^ | 89 (10.5%) | 42 (9.9%) | 47 (11.1%) | 0.654 |
| LDL-c (mg/dl) | 2.67 (2.08-3.15) | 2.67 (2.13-3.23) | 2.66 (2.07-3.12) | 0.608 |
| LVEF (%) | 59.0 (56.0-63.0) | 59.0 (56.0-63.0) | 59.0 (56.0-63.0) | 0.539 |
| Cardiac aneurysm | 13 (1.5%) | 7 (1.7%) | 6 (1.4%) | 1.000 |
| **Concomitant medication---No, %** | | | | |
| Aspirin | 772 (91.0%) | 385 (90.8%) | 387 (91.3%) | 0.904 |
| Clopidogrel/ Ticagrelor | 823 (97.1%) | 410 (96.7%) | 413 (97.4%) | 0.686 |
| DAPT | 753 (88.8%) | 374 (88.2%) | 379 (89.4%) | 0.663 |
| Statin | 835 (98.5%) | 418 (98.6%) | 417 (98.3%) | 1.000 |
| ACEI/ ARB/ ARNI | 493 (58.1%) | 248 (58.5%) | 245 (57.8%) | 0.889 |
| Oral anticoagulant | 17 (2.0%) | 8 (1.9%) | 9 (2.1%) | 1.000 |

Abbreviations: CAD, coronary atherosclerotic heart disease; TIA, Transient Ischemic Attacks; STEMI, ST-segment elevation myocardial infarction; MI, Myocardial infarction; PTCA, percutaneous transluminal coronary angioplasty; PCI, percutaneous coronary intervention; CABG, Coronary Artery Bypass Grafting; SBP, systolic blood pressure; DBP, diastolic blood pressure; CK-MB, creatine kinase isoenzyme MB; eGFR, estimated glomerular filtration rate; LDL, low-density lipoprotein; LVEF, Left ventricular ejection fraction; DAPT, dual antiplatelet therapy; ACEI, Angiotensin-Converting Enzyme Inhibitor; ARB, Angiotensin Receptor Blocker; ARNI, Angiotensin receptor enkephalin inhibitor.

**Table S2.** Short-term risk of cardiovascular outcomes in patients after propensity score matching **^a^**.

| Variables | With beta-blockers | Without beta-blockers |  |  |  |  |
| --- | --- | --- | --- | --- | --- | --- |
|  | N=424 | N=424 | Crude HR | *P*-value | Adjusted HR **^b^** | *P*-value |
|  | No. of patients with event (n, %) | No. of patients with event (n, %) | (95% CI) |  | (95% CI) |  |
| **One year after discharge (n=835)** | | | | | | |
| All-cause mortality | 9/415 (2.2%) | 7/420 (1.7%) | 1.30 (0.48-3.50) | 0.601 | 1.08 (0.40-2.94) | 0.875 |
| Rehospitalization for any reason | 58/404 (14.4%) | 78/415 (18.8%) | 0.74 (0.53-1.04) | 0.087 | 0.71 (0.50-1.00) | 0.050 |
| Cardiac death | 5/415 (1.2%) | 3/420 (0.7%) | 1.69 (0.40-7.07) | 0.473 | 1.35 (0.32-5.71) | 0.680 |
| Rehospitalization for MI | 1/415 (0.2%) | 12/420 (2.9%) | 0.08 (0.01-0.64) | 0.017 | 0.08 (0.01-0.59) | 0.014 |
| Rehospitalization for HF | 9/415 (2.2%) | 4/420 (1.0%) | 2.28 (0.70-7.42) | 0.169 | 2.30 (0.70-7.49) | 0.168 |
| MACE | 11/415 (2.7%) | 19/420 (4.5%) | 0.58 (0.28-1.22) | 0.151 | 0.53 (0.25-1.13) | 0.100 |

This analysis was performed on the post-matched population (N=848) using Univariate Cox analysis and propensity score IPTW correction to determine the relationship between beta-blockers and outcomes in the short-term.

^a^ Abbreviations: MI, myocardial infarction, HF, heart failure, MACE, major adverse cardiovascular events, HR, hazard ratio, NA, not applicable, ref, reference.

^b^ Correction was performed using inverse probability treatment weighting (IPTW), included variables were sex, age, time of onset, LVEF, type of myocardial infarction, admission heart rate, admission systolic blood pressure, admission diastolic blood pressure, body mass index (BMI), site of myocardial infarction, Killip ≥ II, history of hypertension, history of diabetes mellitus, history of chronic kidney disease, history of coronary artery disease (CAD), family history of CAD, history of stroke, history of peripheral vascular disease, history of hyperlipidemia, history of smoking, history of tumor, atrial fibrillation, coronary angiography, PTCA therapy, PCI therapy, thrombolytic therapy, type of PCI, timely reperfusion therapy, total reperfusion therapy, CKMB, TnI, HbA1c, blood urea nitrogen, creatinine, eGFR, LDL-c, cardiac aneurysm, anticoagulants, aspirin, clopidogrel/ticagrelor, dual antiplatelet therapy, statins, ACEI/ARB/ARNI.

**Table S3.** Long-term risk of cardiovascular outcomes in patients after propensity score matching **^a^**.

| Events | With beta-blockers | Without beta-blockers | *P*-value |
| --- | --- | --- | --- |
| **All-cause mortality** | | | |
| No. of patients with event ^a^ | 35/424 (8.3%) | 33/424 (7.8%) |  |
| Unadjusted HR (95% CI) | 0.99 (0.61-1.59) | 1.00 (ref) | 0.964 |
| Adjusted HR (95% CI) ^b^ | 0.96 (0.60-1.55) | 1.00 (ref) | 0.872 |
| **Rehospitalization for any reason** | | | |
| No. of patients with event | 161/413 (39.0%) | 169/419 (40.3%) |  |
| Unadjusted HR (95% CI) | 0.87 (0.70-1.08) | 1.00 (ref) | 0.214 |
| Adjusted HR (95% CI) | 0.86 (0.69-1.07) | 1.00 (ref) | 0.170 |
| **Cardiac death** | | | |
| No. of patients with event | 24/424 (5.7%) | 19/424 (4.5%) |  |
| Unadjusted HR (95% CI) | 1.18 (0.65-2.16) | 1.00 (ref) | 0.585 |
| Adjusted HR (95% CI) | 1.17 (0.64-2.15) | 1.00 (ref) | 0.606 |
| **Recurrent myocardial infarction** | | | |
| No. of patients with event | 12/424 (2.8%) | 18/424 (4.2%) |  |
| Unadjusted HR (95% CI) | 0.61 (0.30-1.27) | 1.00 (ref) | 0.190 |
| Adjusted HR (95% CI) | 0.58 (0.28-1.19) | 1.00 (ref) | 0.135 |
| **Rehospitalization for heart failure** | | | |
| No. of patients with event | 26/424 (6.1%) | 19/424 (4.5%) |  |
| Unadjusted HR (95% CI) | 1.32 (0.73-2.38) | 1.00 (ref) | 0.360 |
| Adjusted HR (95% CI) | 1.35 (0.75-2.44) | 1.00 (ref) | 0.322 |
| **MACE** | | | |
| No. of patients with event | 46/424 (10.8%) | 45/424 (10.6%) |  |
| Unadjusted HR (95% CI) | 0.95 (0.63-1.43) | 1.00 (ref) | 0.797 |
| Adjusted HR (95% CI) | 0.95 (0.63-1.44) | 1.00 (ref) | 0.818 |

The long-term relationship between beta-blockers and outcomes was analyzed. Univariate Cox analysis and propensity score IPTW corrected was performed for patients after propensity score matching (N=848).

**^a^** Abbreviations: AMI, acute myocardial infarction, LVEF, left ventricular ejection infraction, MACE, major adverse cardiovascular events, HR, hazard ratio, ref, reference.

**^b^** Correction was performed using inverse probability treatment weighting (IPTW), included variables were sex, age, time of onset, LVEF, type of myocardial infarction, admission heart rate, admission systolic blood pressure, admission diastolic blood pressure, body mass index (BMI), site of myocardial infarction, Killip ≥ II, history of hypertension, history of diabetes mellitus, history of chronic kidney disease, history of coronary artery disease (CAD), family history of CAD, history of stroke, history of peripheral vascular disease, history of hyperlipidemia, history of smoking, history of tumor, atrial fibrillation, coronary angiography, PTCA therapy, PCI therapy, thrombolytic therapy, type of PCI, timely reperfusion therapy, total reperfusion therapy, CKMB, TnI, HbA1c, blood urea nitrogen, creatinine, eGFR, LDL-c, cardiac aneurysm, anticoagulants, aspirin, clopidogrel/ticagrelor, dual antiplatelet therapy, statins, ACEI/ARB/ARNI.


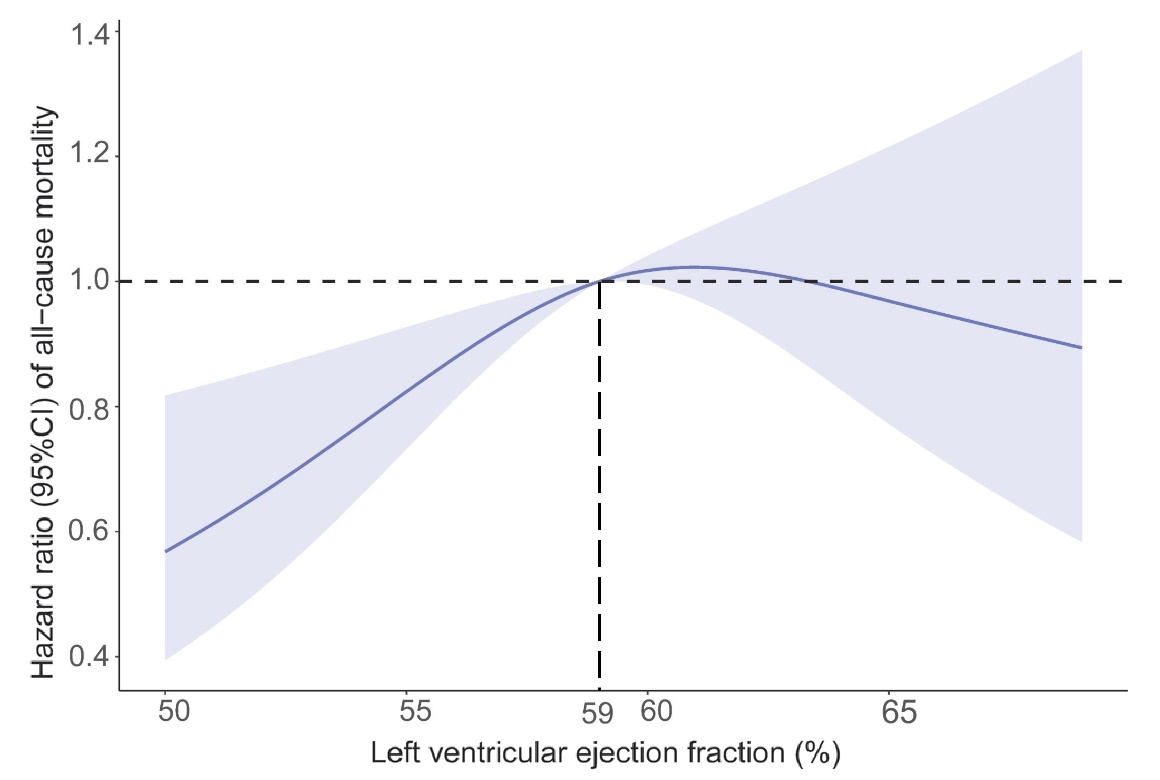


**Figure S1.** Hazard ratios (HR) for all-cause mortality, adjusted using IPTW, according to LVEF using restricted cubic spline (RCS). The shaded areas represent the 95% Confidence interval. The scale on the y-axis indicates HR for all-cause mortality, where values more than one indicate the greater rate of all-cause mortality and values less than one indicate fewer all-cause mortality are related to an LVEF on the x-axis. IPTW correction: based on propensity score with IPTW, adjusted for factors as described in Table 2.
